# Bioacoustic IoT Sensors as Next-Generation Tools for Monitoring: Counting Flying Insects through Buzz

**DOI:** 10.3390/insects14120924

**Published:** 2023-12-05

**Authors:** Simona Alberti, Gianluca Stasolla, Simone Mazzola, Luca Pietro Casacci, Francesca Barbero

**Affiliations:** 1 Department of Life Sciences and Systems Biology, University of Turin, Via Accademia Albertina 13, 10123 Turin, Italy; francesca.barbero@unito.it; 2 Independent Researcher, 70029 Santeramo in Colle, Italy; stasollag@gmail.com; 3 3Bee srl, Via Alessandro Volta 4, 20056 Trezzo Sull’Adda, Italy; simone.mazzola@3bee.com

**Keywords:** innovative technology, Internet of Things, bioacoustic monitoring, traditional methods, pan traps, pollinators, biodiversity

## Abstract

**Simple Summary:**

The decline in global biodiversity due to habitat loss, climate change, and human activities necessitates effective monitoring. Flying insects, such as pollinators, which are crucial for ecosystem functions, require a thorough assessment. While informative, conventional methods, including hand netting, transect surveys, and pan traps, are labour-intensive or offer snapshots of limited scope. This study conducts a comparative analysis of traditional and innovative methods involving the recording and analysis of buzzing sounds emitted by flying insects. Insect abundance, reported as counts for traditional methods gathered by pan traps, and average buzzes per hour, measured by acoustic IoT (Internet of Things) sensors, were correlated. This finding suggests that acoustic automatic monitoring shows the potential to circumvent the limitations of conventional methods, emphasising continuous, non-intrusive monitoring for robust data acquisition. The development of passive detection devices will pave the way to implement new, sustainable approaches to enhance conservation efforts and ecosystem management, advancing our understanding of flying insect dynamics amidst global biodiversity challenges.

**Abstract:**

The global loss of biodiversity is an urgent concern requiring the implementation of effective monitoring. Flying insects, such as pollinators, are vital for ecosystems, and establishing their population dynamics has become essential in conservation biology. Traditional monitoring methods are labour-intensive and show time constraints. In this work, we explore the use of bioacoustic sensors for monitoring flying insects. Data collected at four Italian farms using traditional monitoring methods, such as hand netting and pan traps, and bioacoustic sensors were compared. The results showed a positive correlation between the average number of buzzes per hour and insect abundance measured by traditional methods, primarily by pan traps. Intraday and long-term analysis performed on buzzes revealed temperature-related patterns of insect activity. Passive acoustic monitoring proved to be effective in estimating flying insect abundance, while further development of the algorithm is required to correctly identify insect taxa. Overall, innovative technologies, such as bioacoustic sensors, do not replace the expertise and data quality provided by professionals, but they offer unprecedented opportunities to ease insect monitoring to support conservation biodiversity efforts.

## 1. Introduction

The global decline in biodiversity has become an alarming concern [1,2,3], with ecosystems facing unprecedented challenges due to habitat loss, climate change, and various anthropogenic activities [4,5,6]. Information on the overall status of the environment and its biodiversity is often obtained through monitoring of a few selected bioindicators [7,8,9]. Among those, pollinators, such as bees, hoverflies, and butterflies, are essential in the food chain and are an integral part of agricultural systems (about 70% of food crops worldwide depend on insect pollination [10]), performing numerous ecosystem services for humans [11]. Indeed, in addition to being essential to plant pollination [12,13], insects contribute to the nutrient cycle, preserve soil structure, spread seeds, and regulate the populations of other organisms [14].

Several flying insects, such as butterflies, wild bees, Diptera, and Coleoptera, are undergoing a sharp decline [15,16,17,18]. However, global change impacts insect species differently, highlighting the existence of “winners”—increasing in abundance or expanding the distributional range—along with “losers”—declining or facing local extinctions [18]. In both cases, it is crucial to implement the proper methodological approach to achieve robust inferences on population trends. Traditional monitoring techniques, such as transect surveys and pan traps, have long been employed to assess insect population dynamics [19]. While these methods provide valuable insights, they are often labour-intensive, resource-demanding, and geographically constrained [20]. Traditional active monitoring methods, such as hand netting or visual transects, supply priceless information that enables accurate assessments of species richness. However, they demand significant sampling efforts. In contrast, traditional passive methods involve lower sampling effort but require the sacrifice of several individuals and exhibit selectivity towards specific flying insect groups [19]. Moreover, the sampling frequency of traditional protocols may result in collecting data that only represent a snapshot of insect activity within a specific timeframe, making it difficult to see short-term fluctuations driven by external variables, such as weather and land-use practices [21]. Indeed, the correlation between insect activity and weather conditions has been extensively investigated, suggesting a thermally-dependent modulation of insect behavioural patterns [22,23,24,25]. In the colder, middle seasons, a marked increase in insect activity is reported during the warmer midday periods [26]; for example, in social bees [27,28,29], flight activity peaks between 20 °C and 25 °C. In hotter seasons, insect activity increases as temperature rises but declines during extreme midday heat [23], revealing a threshold beyond which elevated temperatures negatively affect insect mobility. On the other hand, adverse weather conditions, particularly on days with significant rainfall, are associated with a drastic reduction in insect activity, often causing its complete cessation [26]. Several factors also drive differences in night and day activities, as well as significant changes in insect diversity. Rosenthal (2004) [30] shows that circadian variations in insect species richness might be affected not only by the effectiveness of trap catching but also by temperature fluctuations between day and night. Several vital metabolic activities could be inhibited or deterred at lower night temperatures [31]. Moreover, diurnal insects exploit sunrays to prompt activities, while nocturnal species exclusively depend on stored energy [31,32]. However, the relative species abundance between night and day varies across taxa as well as their biological traits (predatory, herbivorous, parasitic) and might be affected by slight temperature changes [30]. At present, traditional methods for evaluating insect diversity may not comprehensively capture the dynamics of insect population fluctuations, both on an intraday and a long-term scale.

The advent of remote sensing technologies and monitoring through images or sound automatic detection are promising alternatives to conventional methods, enabling comprehensive and continuous data collection and overcoming some of the limitations of traditional approaches [33]. Remote sensing, coupled with satellite imagery and spatial modelling, can provide valuable information on landscape characteristics and environmental variables that influence insect activity [33,34,35,36]. Image processing and neural network analysis were employed to identify orchard insects [37] and Lepidoptera [38] successfully. On the other hand, Passive Acoustic Monitoring (PAM), which encompasses real-time analysis of data collected by acoustic IoT (Internet of Things) sensors, is becoming an increasingly important tool of ecological research [39] used in various types of surveys, from autonomous bird species recording [40] to urban traffic noise monitoring [41]. Due to the low cost, ease of installation, efficiency, and energy independence of these acoustic devices, it is possible to facilitate real-time and large-scale monitoring [42], acquiring important information on population diversity and dynamics, over a finer daily scale or more extended periods to complement traditional methods. Data processing includes identification algorithms to detect the presence of species within an environment, recognising distinct sounds and inferring species diversity. Previous studies have already demonstrated the importance of sound detectors in recognising insects based solely on the sounds produced by the wings during flight [43,44,45,46,47,48].

In this work, we explore the potential of insect monitoring conducted by IoT bioacoustic sensors as an innovative approach to assess biodiversity changes, focusing on flying insect populations. We present a comparative analysis of data collected using traditional monitoring methods (hand-netting surveys and pan traps) and IoT bioacoustic sensors. Based on PAM, we obtained reliable estimates of flying insect abundance. Therefore, our results highlight the benefits of employing bioacoustic sensors in assessing the temporal variation and abundance of flying insects, providing a continuous and non-invasive assessment of insect population dynamics. We believe that the further development of these new technologies will pave the way for PAM to become one of the most affordable, widespread, and reliable approaches in insect monitoring, ultimately providing essential insights for biological conservation and ecosystem management strategies.

## 2. Materials and Methods

### 2.1. Study Areas

To carry out the comparative analysis between PAM and traditional methods, four farms located in Italy with different characteristics, locations, and crop productions were chosen:

- Site A→located in northern Italy in the municipality of Grezzana (VR), Veneto (45.516667°, 11.016667°); a very diversified farm of about 40 ha, with land mainly used for wine production, smaller areas of olive groves, and deciduous woodland. Annual temperatures range from −7 °C to 37 °C (9–32 °C between mid-April and mid-June 2023), and monthly precipitation between 30 mm and 77 mm (58–62 mm between mid-April and mid-June 2023).
- Site B→located in central-northern Italy in the municipality of Medicina (BO), Emilia-Romagna (44.483333°, 11.633333°); 30 ha of arable crops surrounded by similar agricultural landscape. Annual temperatures range from −5 °C to 38 °C (7–34 °C between mid-April and mid-June 2023), and monthly precipitation between 27 mm and 66 mm (40–46 mm between mid-April and mid-June 2023).
- Site C→located in central Italy in the municipality of Viterbo (VT), Lazio (42.418611°, 12.104167°); an area of approximately 2 ha used for growing hazelnuts (*Corylus avellana*), surrounded by a dirt road with spontaneous aromatic flowers and other hazelnut groves. Annual temperatures range from −1 °C to 37 °C (8–29 °C between mid-April and mid-June 2023), and monthly precipitation between 18 mm and 86 mm (30–45 mm between mid-April and mid-June 2023).
- Site D→located in central Italy in the municipality of Viterbo (VT), Lazio (42.421027°, 12.129143°); an area of approximately 1 ha where half is used for the cultivation of hazelnuts (*Corylus avellana*) while the other half has been left to spontaneous re-naturalisation; it is set in a broadleaf woodland context (chestnut and beech woods). Annual temperatures range from −1 °C to 37 °C (8–29 °C between mid-April and mid-June 2023), and monthly precipitation between 18 mm and 86 mm (30–45 mm between mid-April and mid-June 2023).

In each of these study areas, bioacoustic sensors (see Section 2.2) were installed in mid-April 2023, and records were collected up to mid-June 2023. During the installation and subsequent removal of these devices, traditional monitoring was conducted by hand-netting transects and pan traps (Table 1). Within each site, all sampling methods were performed in the same habitat, light conditions, and canopy coverage. In detail, at Sites C and D, acoustic devices and traditional methods were placed in the hazel grove; at Site B, the environment was homogenous and open; at Site A, both PAM and traditional methods were carried out in the vineyard and in the olive grove.

The number of replications using traditional methods was established according to the type and size of each study area, and the number of bioacoustic devices deployed at each site was equivalent to the number of replicates, 16 in total. All acoustic devices were placed within 200 metres from the hand-netting transects and pan traps. Overall, the three sampling methods were performed within a 400 m radius of unfragmented and uniform habitats. This range is commonly covered by insects flying in agricultural landscapes [49].

### 2.2. Spectrum

The bioacoustic IoT sensor used in this study is called Spectrum and was conceived and developed by the Italian company 3Bee Srl [50]. Spectrum is equipped with an IM73A135V01 microphone designed for applications requiring high SNR (Signal to Noise Ratio), low distortion (high AOP), and IP57 dust and water resistance. It features a 6–9 Volt polycrystalline solar panel connected to the batteries via a USB type C cable that can recharge the device autonomously. Recordings were made from 6:00 to 21:00 with a duty cycle of 20% (12 min on and 48 off) and from 21:00 to 6:00 with a duty cycle of 7% (4 min on and 56 min off). This schedule yields a total of 216 min of recording per day, maximising the effectiveness of sampling within the batteries’ energy budget.

The recordings made by Spectrum are stored and then uploaded to the cloud database via LTE (Long Term Evolution) using a multi-operator sim. This allowed further data processing based on sound bursts’ spectral content. An algorithm recognises the buzz in the recorded audio track and isolates it as a single event, producing an output file with the number of buzzes, hereafter called “counts”.

**Figure 1:**
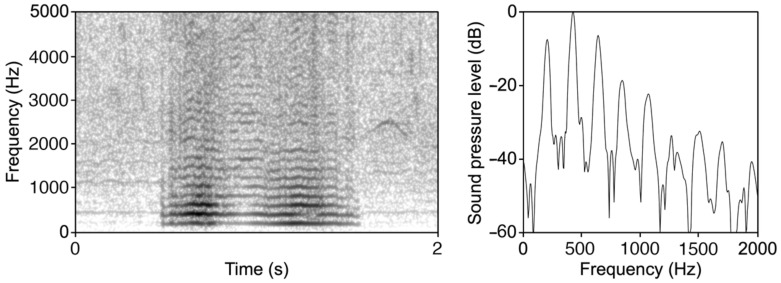
Example spectrogram and power spectrum of the wing buzzing of *Bombus pratorum*. The spectrogram was generated in Praat version 6.2.14 using a Gaussian window shape, a window length of 0.03 s, 1000 time steps, 250 frequency steps, and a dynamic range of 70 dB. The power spectrum was generated by selecting a portion of 0.01 s of the signal where the frequency components contain the highest energy.

The event classification is performed using the following steps:

1. Acoustic Sensor Acquisition: The device acquires the sound spectrum between 4 Hz and 3 kHz in real time, a range covering most of insect buzzing frequencies, such as Hymenoptera, Diptera, and Orthoptera [44,45,47,48,51,52,53,54,55];
2. Fourier Transformation: The captured signal undergoes Fourier Transformation at a resolution of 5 Hz, converting it from the time domain to the frequency domain;
3. Buzz Pattern Identification: The transformed data are then processed by an algorithm to identify and enumerate patterns classifiable as ‘buzz’. The algorithm detects the fundamental frequency and checks for subsequent peaks at higher harmonics (see Figure 1).

The accuracy of the event count was assessed by manual verification and random listening of 3% of the recordings identified as buzz; for 1146 events analysed out of the total 46,632, 76% of the events were considered valid, 22% in doubt, and 2% wrong.

### 2.3. Invertebrate Surveys: Traditional Methods

To document the invertebrate fauna, hand collection of adults with an entomological net [56] and pan traps [57] was implemented in April and June 2023 (Table 1). Collection of invertebrates was conducted by experienced people using a hand net (with a 30 cm diameter opening) at each of the four sites. Linear 50 m long transects performing a sweep for each step were carried out during sunny days (from 10:00 to 16:00). The adults were transferred into a killing jar and later pinned and labelled. In total, we surveyed 16 transects during each period among the four sites.

Pan traps used to collect flying insects were painted in one of the following UV-reflecting colours: blue, white, yellow, or red (Sparvar Leuchtfarbe, Spray-Color GmbH, Merzenich, Germany). The pans had a diameter of 20 cm and were filled with a solution of 2.5 mL of soap and 2 L of water. Pan trap sets were placed at the same height as the vegetation and in places that were considered representative of different habitats/crops. In total, 16 sets of pan traps (equal numbers of blue, white, yellow, and red items) were left out during each period at the four sites for 4 h during sunny days (from 10:00 to 16:00); after sampling, trapped insect specimens were removed by forceps and placed in ethanol.

Adult specimens were sorted into RTUs (Recognizable Taxonomic Units) [58,59,60,61] in the laboratory using a binocular microscope. These identifications allowed us to compare data gathered with traditional methods and acoustic sensors by considering the whole community or by creating subsets, which included only the flying insects. The latter have been defined for this analysis as all those insects that have a winged phase in at least one stage of their life cycle (Hymenoptera, Diptera, Hemiptera, Orthoptera, Lepidoptera, and Coleoptera). All samples are deposited in the collection of G.S. in Santeramo in Colle (BA), 70029, Italy. Only rough total abundances of specimens were used in further analysis.

### 2.4. Statistical Analysis

The average buzz rate per hour (buzz/h) was calculated per device at each site by considering only the recordings between 10:00 and 16:00 to be consistent with the time slot set by the traditional methods. The total number of buzzes recorded in this limited time frame was divided by the total minutes of recording (12 min per hour) and then converted to hours. Data collected using traditional methods were standardised by dividing the total abundance of individuals by the number of replicates.

Normality of data was tested using the Kolmogorov–Smirnov test, and thereafter parametric or nonparametric tests were applied accordingly. The nonparametric Kruskal –Wallis test was used to evaluate the differences in bioacoustic sensors’ buzz/h among sites, while the differences in the standardised animal counts from hand netting and pan traps were tested using ANOVA (function *aov* from R package stats) and Tukey post hoc test (function *TukeyHSD* from R package stats). We estimated the Pearson correlation coefficients (r) and their significance (*rcorr* function from the R package Hmisc [62]) between (i) the bioacoustic sensors’ buzz/h and the standardised animal counts gathered with pan traps; (ii) the bioacoustic sensors’ buzz/h and the standardised counts of flying insects gathered with pan traps; (iii) the bioacoustic sensors’ buzz rate per hour (buzz/h) and the standardised animal counts derived from hand netting; and (iv) the standardised animal counts from hand netting and pan traps.

At Site A, we used data loggers to record air temperature variations throughout the bioacoustic sensors’ recording period. We randomly selected three dates showing medium-low (8.6–17.6 °C) and medium-high temperatures (18.4–30.4 °C) and a rainy day. We calculated the average hourly buzzes among the four bioacoustic devices located at Site A from 6:00 to 21:00 to assess whether the variation in buzz/h was consistent with insect activity changes (e.g., lower activity during rainy days [26]) due to weather conditions. Similarly, we calculated the average hourly buzzes for each device for all recording dates to see the distribution in temperature ranges over the period.

To show the variability in insect activity over time, we calculated hourly average buzzes for each device at Site A as described earlier, considering daytime (6:00 to 21:00) and the whole 24 h over a two-month period.

For graphs, we used the *ggplot2* package [63]. All analyses were carried out in R v. 4.2.2 [64].

## 3. Results

### 3.1. Data Analysis

**Figure 2:**
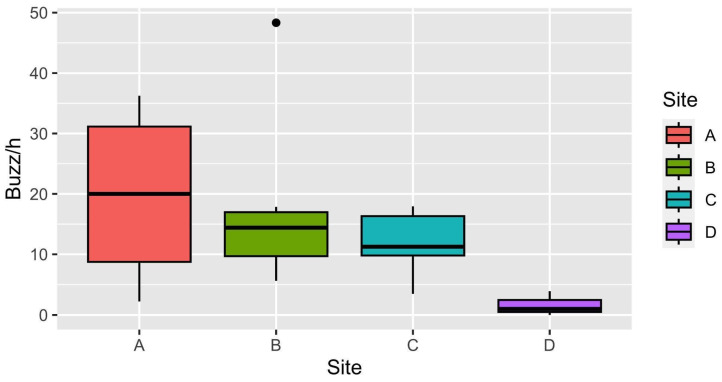
Boxplots of number of buzzes per hour of acoustic sensors calculated per site, considering only the recordings between 10:00 and 16:00 in April and June. Horizontal lines represent median rank of values, boxes indicate 25th and 75th percentiles, whiskers correspond to minimum and maximum values, and dots represent outliers.

**Figure 3:**
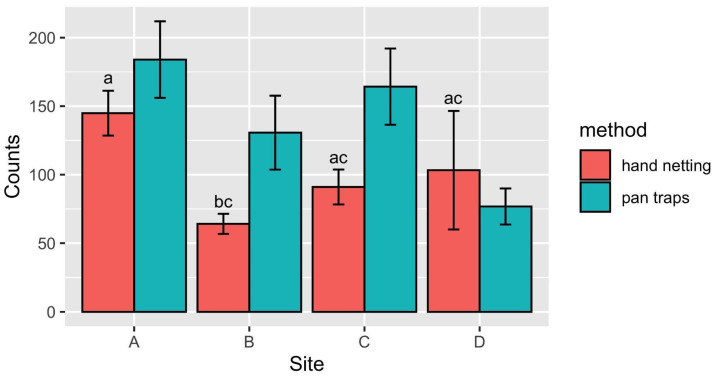
Mean (±SE) insect abundance per site in April and June. Insect abundance in pan traps did not differ between sites. Sites with different letters had significantly different numbers of insects in hand nets (based on the Tukey test).

The distribution of insect abundance across the study sites is reported in Figure 2 and Figure 3. This initial step helps us understand how insect populations vary among these sites, considering acoustic data (Figure 2) and those of traditional methods (Figure 3). Using hand netting, we collected invertebrates, mostly insects, while the pan traps were selective only for flying insects (captures of non-flying specimens are anecdotal).

Between study sites, we observed only slight differences in insect abundance, measured as the number of buzzes per hour by bioacoustic sensors (H = 6.941; d.f. = 3; P = 0.074). On average, Site A exhibited the highest insect median abundance, followed closely by Sites B and C, while Site D showed the lowest values (Figure 2).

Considering the total abundance obtained by hand netting and pan traps, on average, Site A showed the highest abundance of sampled individuals, followed by Sites C and B. As revealed by acoustic sensor data, Site D showed the lowest insect abundance. When considering only pan traps, Site A was the site with the highest average abundance, followed by Site C, Site B, and Site D (Figure 3). Also considering only hand netting, Site A showed the highest average abundance, followed, in order, by Sites D, C, and B (Figure 3). However, differences among sites were only significant for hand netting (F_Hand netting 3,32_ = 5.523; P = 0.004; F_Pan traps 3,32_ = 1.824; P = 0.166), with Site A and Site B being significantly different (Tukey post hoc test, P = 0.002).

To investigate the correlation between insect abundance estimated by traditional sampling methods, specifically hand netting and pan traps, and the use of bioacoustic sensors, we performed the analysis at two distinct time points, April and June, across four different study sites, resulting in a total of eight data points.

**Figure 4:**
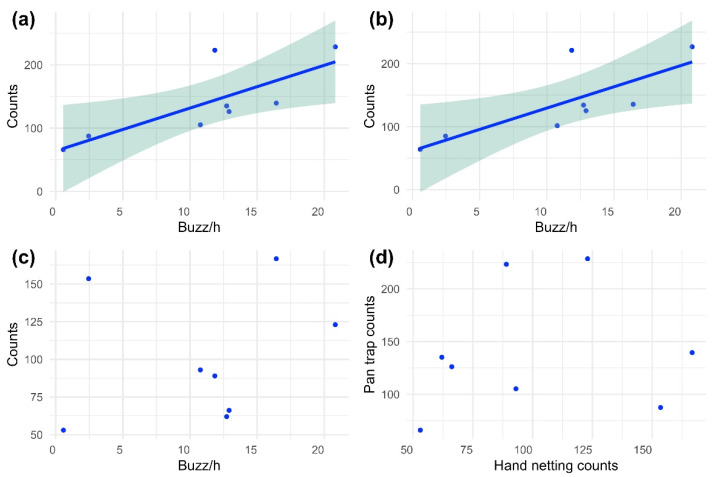
(**a**) Scatterplot of standardised animal counts, focusing exclusively on pan trap data, and bioacoustic sensors’ buzz rate per hour (buzz/h). (**b**) Scatterplot of standardised animal counts, focusing exclusively on flying insects from pan trap data, and bioacoustic sensors’ buzz rate per hour (buzz/h). (**c**) Scatterplot of standardised animal counts, focusing exclusively on hand netting data, and bioacoustic sensors’ buzz rate per hour (buzz/h). (**d**) Scatterplot of standardised animal counts of hand netting data and pan trap data. The coloured bands represent a 95% confidence interval for estimating the regression line (in blue).

The only significant correlation between traditional and acoustic sampling was with pan traps. The correlation was positive and significant when we considered both the whole standardised animal counts (r = 0.768, N = 8, P = 0.026; Figure 4a) and the standardised counts of flying insects (r = 0.766, N = 8, P = 0.027; Figure 4b). On the contrary, when examining buzz rate per hour (buzz/h) against animal counts standardised by the number of replications for hand netting, we observed a positive, although not significant, correlation (r = 0.240, N = 8, P = 0.567; Figure 4c). Moreover, when considering the standardised counts of only flying insects collected by hand netting, the correlation was positive, but low and not significant (r = 0.210, N = 8, P = 0.618). Comparing the abundance yielded by the two traditional sampling methods, hand netting and pan traps, the correlation was found to be weak and not significant (r = 0.150; N = 8; P = 0.722; Figure 4d).

### 3.2. Hourly and Weekly Variation of Buzzes

On the day with medium-low temperatures, the range was between 8.6 °C and 17.6 °C, while on the day with medium-high temperatures it was between 18.4 °C and 30.4 °C. On the rainy day, there was 34.4 mm of rain, and temperatures were between 12.1 °C and 15.7 °C (Table 2).

**Figure 5:**
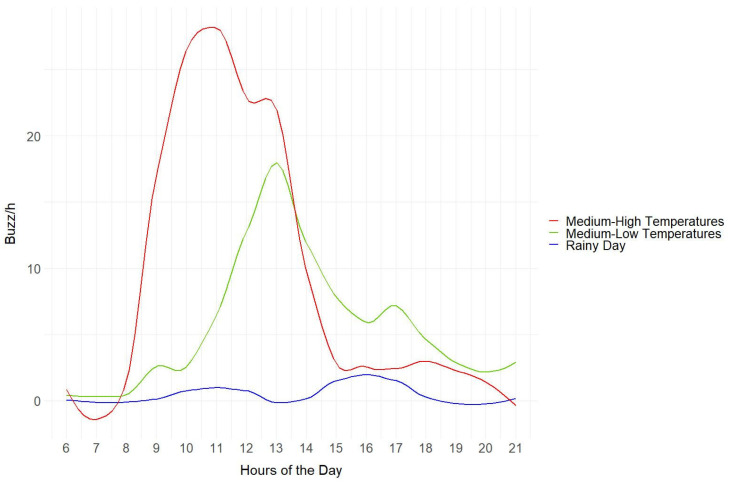
Daily variation in the average number of buzzes per hour of the four bioacoustic sensors at Site A from 6:00 to 21:00.

On days with medium-low temperatures, the buzzes were more concentrated in the central hours of the day (11:00–17:00) when temperatures rose to 17.6 °C. In contrast, on days characterised by medium-to-high temperatures, we recorded a higher number of buzzes, but earlier in the day (8:00–15:00). Early afternoon buzzes then started to decrease (from 13:00) because it became too hot (30.4 °C) (Figure 5). On rainy days, buzzes were close to zero (Figure 5).

**Figure 6:**
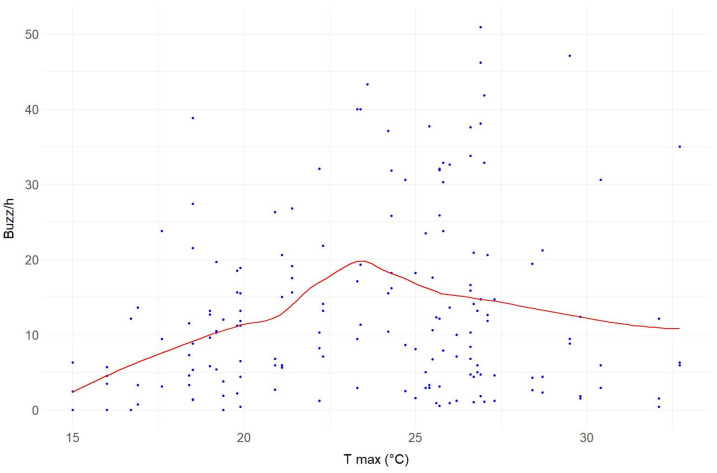
Distribution of buzz/h over the temperature ranges recorded at Site A during the two months of sampling. The *y*-axis represents the buzz/h of the four devices at the site, and the *x*-axis represents the maximum temperature recorded during the reference period. The red line is the trend line obtained by the “loess” method.

The mean hourly buzz activity corresponding to each temperature range measured continuously over the two-month sampling period at Site A is reported in Table 3 The highest activity was recorded between 20 °C and 25 °C, followed shortly by the range between 25 °C and 30 °C, and then the activity dropped below 20 °C and above 30 °C (Figure 6).

**Figure 7:**
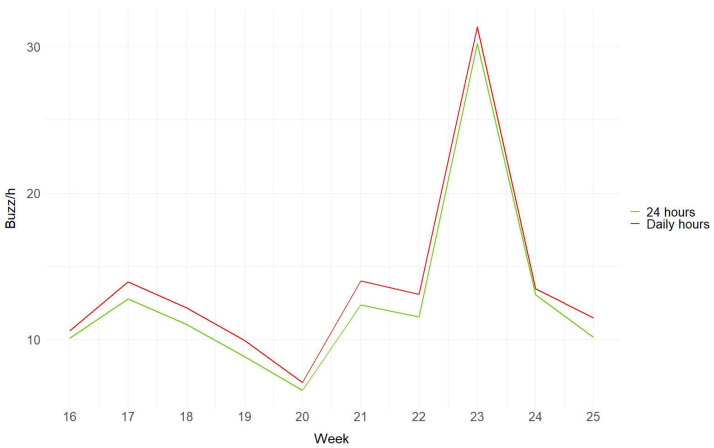
Trend of buzzing activity for Site A, showing buzz/h during all 24 h and only the hours of the day (6:00 to 21:00). The *x*-axis represents weeks during the two months of recording from mid-April (spring) to mid-June (summer), while the *y*-axis represents the average buzz per hour per week.

Extending the recording time over the 24 h period did not significantly increase the average number of buzzes (Mann–Whitney U_20,1_ = 64; P > 0.05; mean_24h_ ± SE = 12.65 ± 6.48; mean_daily_ ± SE= 13.70 ± 6.56) (Figure 7).

## 4. Discussion

Recent evidence of biodiversity loss and declining insect populations urgently calls for more effective species conservation strategies [65], which nowadays can be implemented thanks to the emergence of new technologies [66,67,68]. Achieving this goal requires advanced tools and skilled professionals to collect reliable and robust qualitative and quantitative data effectively, thus facilitating more targeted assessments [69]. Our findings underscore the usefulness and benefits of innovative bioacoustics-based devices as a viable data source in biodiversity monitoring. Specifically, we have identified a positive correlation between acoustic data and pan traps (Figure 4). Despite being based on a limited number of comparisons, this correlation proved to be robust (correlation coefficient > 0.7), highlighting the potential effectiveness of IoT sensors in assessing the abundance of flying insects without causing any invasive impact on wildlife, in contrast to pan traps [70]. The poor correlations between the data collected by the two passive monitoring methods (bioacoustic sensor and pan traps) and hand netting suggest that the communities censused by the traditional active method of hand netting encompass distinct taxa [71]. However, when we analysed data subsets that included only flying species, we found no substantial increase in the correlation coefficient. These results indicate that hand netting sampling is biased towards insect groups with restricted ecological requirements dissimilar to those captured by pan traps and PAM, perhaps occurring in a narrower niche or emitting buzzing at undetectable frequencies [72,73]. Therefore, even if our results suggest a similar trend in insect abundance estimated by hand netting and acoustic sensors, PAM needs further development to be considered an alternative method to sweeping. In contrast, the time seems ripe to start assessing insect abundance with PAM, thus avoiding the killing of individuals as it occurs when pan traps operate.

Furthermore, we demonstrated the suitability of this device for intraday and long-term monitoring. Indeed, by examining a specific site, we showed that daily variations in buzzes detected by bioacoustic sensors align with insect activity patterns based on weather conditions, confirming the strong influence of temperature on insect activity (Figure 5 and Figure 6). Our results are consistent with the findings by Ma and colleagues [22] and other studies on insects [23,24,25], demonstrating that insect activity is concentrated during the warmer midday hours on days with moderate temperatures [26]. We observed that, as temperatures rise, insect activity increases accordingly but declines during the hottest midday hours [23], while on rainy days insect activity is virtually nonexistent [26]. Consistent with the expectation of higher mobility of diurnal insects [30,31,32,74], the daily trends at Site A showed an increase of around 20% in the activity detected with buzz/h during daylight hours. When analysing the acoustic data collected over a two-month period, we observed an increase in buzzing within the temperature range of 20 °C to 25 °C; conversely, there was a decrease in activity when temperatures fell below 20 °C or rose above 30 °C (Figure 6). This temperature-dependent pattern aligns with previous findings describing the variation in the activity in certain social bees [27,28,29]. It should be noted, however, that having removed the bioacoustic sensors in mid-June, the days with maximum temperatures above 30 °C were few. Therefore, this type of survey should be extended over a longer period to support our results. The study of daily and weekly variations in acoustic buzz provides insightful observations into the dynamic patterns of flying insect activity, revealing significant fluctuations in their presence from day to day (Figure 7). With their sensitive and continuous detection capabilities, devices used in PAMs, as presented in this study, can capture minute variances in insect buzz frequencies, offering a more nuanced understanding of insect behaviour and population dynamics over time. In contrast, this remarkable temporal variability reveals a considerable potential for bias in traditional samplings based on fixed-frequency monitoring events. This capability is crucial for ecological research, as it allows for the long-lasting and non-invasive monitoring of insect populations, providing valuable data that can inform conservation strategies and ecological assessments, eventually detecting variations that can be correlated to global warming or climate change.

Even though the acoustic devices already available allow the recording of a wide variety of taxa, including Diptera, Hymenoptera, Orthoptera, Hemiptera, and Coleoptera [45,47,51,52,53,54,55,72,73,75,76], our current approach does not enable biodiversity estimation, as already implemented for crickets and birds [77,78,79,80,81]. Traditional active methods, such as hand netting or visual transects, remain the best tools for assessing insect species richness and local biodiversity but require extensive sampling efforts [82]. Therefore, to fill this gap, a crucial development of devices employed in PAM should consider improving a recognition algorithm to identify flying insect taxonomic units correctly. Studies aiming at dissecting differences in insects’ acoustic patterns have already shown species-specific variations in some sound parameters or combinations of them, such as for bumblebees [44], bees, and hornets [45,47] or crickets, cicadas, and katydids [46]. The identification of insects, such as wild bees and hoverflies, by passive acoustic methods is already possible for some species [44,45,47], and the potential for extending PAM broadly and to other taxa is enormous. The applicability of this monitoring technique in conservation initiatives is underscored by its nondestructive approach, making it well-suited for studying endangered and protected insect species. This method facilitates the collection of data regarding their presence and activities, negating the need for invasive practices, such as sacrificing individuals for identification purposes. This aspect not only ensures the safety and preservation of vulnerable populations but also provides a more ethical and sustainable approach to ecological research and species conservation.

Nevertheless, most studies implementing PAM have focused on a few target species using existing or newly originated algorithms that have taken a moderate amount of time to be devised. In contrast, a more comprehensive method capable of identifying a wider range of species belonging to different orders remains undeveloped. Implementing highly sensitive dual microphones, along with optimised hardware and an improved signal-to-noise ratio (SNR) to minimise interference and ambient noise, could be the key achievements to be pursued. Still, as pointed out by Abdollahi and colleagues [83], in addition to technical constraints, there are several aspects to consider in the process of creating an automatic recognition algorithm, including the need for interdisciplinary collaborations with taxonomists to provide accurate species identification, physicists to set replicable recording parameters for creating the sound library, and mathematicians to choose the best training configuration. We believe that at the current rate of technological development, within a decade, acoustic devices and algorithms will be commonly employed as a stand-alone method to assess insect biodiversity across a wide variety of habitats.

A possible limitation of our study is the number of environmental variables that may have influenced our results, as, for example, the level of background noise that can conceal buzzing insects, including winds and agricultural machinery. Another constraint is the number of sites and sampling days considered for comparing traditional and innovative approaches, which could have led to partial conclusions, especially when comparing hand netting and acoustic device data. In future advancements, expanding the study to encompass a larger number of sites and more extensive sampling periods is highly recommended. This broader approach will facilitate in-depth analyses, enabling a more profound comprehension of the fluctuations and responses exhibited by the local flying insect populations’ activities.

## 5. Conclusions

In this paper, we showed the potential of adopting innovative technologies to monitor local biodiversity, paving the way for the development of similar tools for environmental conservation. The implementation of new technologies for ecology and insect monitoring will complement, rather than replace, specialised taxonomic knowledge and traditional methods, which undeniably provide qualitatively informative data of value. However, traditional methods require extensive sampling efforts, the sacrifice of many specimens, and may be strongly affected by the weather changes during the sampling [20].

In conclusion, while these innovative methodologies do not replace the expertise and data quality provided by professionals, they offer unprecedented opportunities for insect research and monitoring in support of conservation and biodiversity efforts.

## Figures and Tables

**Table 1:** Sampling periods for each site, number of replicates per method (HN = hand netting transect; PT = pan traps set; BS = bioacoustic sensors) and habitat types with the respective number of sampled areas reported in parentheses.

Site	Date	Method			Habitat
		HN	PT	BS	
Site A	22–25/04/2023	4	4	4	Vineyard (3); olive grove (1)
	19–22/06/2023	4	4	4	
Site B	21–24/04/2023	6	6	6	Arable crops (6)
	18–21/06/2023	6	6	6	
Site C	12–15/04/2023	4	4	4	Hazelnut grove (4)
	17–20/06/2023	4	4	4	
Site D	12–15/04/2023	2	2	2	Hazelnut grove (1); re-naturalised hazelnut grove (1)

**Table 2:** Dates, minimum and maximum temperatures, and millimetres of rainfall of the three selected days at Site A.

Date	Variable	T Min (°C)	T Max (°C)	Rain (mm)	Sunrise Time	Sunset Time
26/04/2023	Medium-Low Temperature	8.6	17.6	0	6:12	20:14
10/05/2023	Rainy day	12.1	15.7	34.4	5:51	20:32
26/05/2023	Medium-high Temperature	18.4	30.4	0	5:34	20:50

**Table 3:** Temperature ranges and mean buzz/h (mean ± SE) found in the two sampling months at Site A (mid-April, mid-June, N = 61 days).

T Max (°C)	Mean Buzz/h ± SE
15–20	8.72 ± 1.11
20–25	17.30 ± 1.86
25–30	16.53 ± 2.06
30+	11.16 ± 4.25

## Data Availability

Data are available upon request to the corresponding authors.
